# 多西他赛联合PD-1/PD-L1抑制剂二线治疗晚期非小细胞肺癌的临床分析

**DOI:** 10.3779/j.issn.1009-3419.2021.102.26

**Published:** 2021-09-20

**Authors:** 青 陈, 艳 李, 文杰 张, 圣杰 杨, 晨 王, 其森 郭, 文娜 史

**Affiliations:** 1 250117 济南，山东第一医科大学附属山东省肿瘤医院肿瘤内科，山东第一医科大学 Department of Oncology, Shandong Cancer Hospital and Institute, Shandong First Medical University and Shandong Academy of Medical Sciences, Jinan 250117, China; 2 261000 潍坊，潍坊医学院 Weifang Medical University, Weifang 261000, China; 3 250117 济南，山东省肿瘤医院药学部 Department of Pharmacy, Shandong Cancer Hospital and Institute, Jinan 250117, China

**Keywords:** 肺肿瘤, 多西他赛, PD1/PD-L1抑制剂, 安全性, 近期疗效, Lung neoplasms, Docetaxel, PD-1/PD-L1 inhibitor, Safety, Efficacy

## Abstract

**背景与目的:**

程序性死亡受体1（programmed cell death 1, PD-1）/程序性死亡配体1（programmed cell death ligand 1, PD-L1）抑制剂和多西他赛作为晚期非小细胞肺癌（non-small cell lung cancer, NSCLC）患者的标准二线治疗方案，治疗效果有限。多西他赛联合PD-1/PD-L1抑制剂是否能增加疗效并使患者更好地获益少有报道。本研究旨在探索多西他赛联合PD-1/PD-L1抑制剂二线治疗驱动基因阴性的Ⅳ期NSCLC的临床疗效和安全性。

**方法:**

选取2018年10月1日-2020年12月31日在山东省肿瘤医院就诊的Ⅳ期NSCLC患者118例，依据治疗方法不同分为观察组（*n*=69）和对照组（*n*=49），观察组患者接受多西他赛联合PD-1/PD-L1抑制剂治疗，对照组患者接受PD-1/PD-L1抑制剂治疗，比较两组患者的临床疗效及3级以上不良反应发生情况。

**结果:**

观察组疾病控制率（disease control rate, DCR）（89.9%）高于对照组（73.5%）（*P*=0.019），客观缓解率（objective response rate, ORR）（24.6%）与对照组（16.3%）比较差异无统计学意义（*P*=0.276）。随访至2021年6月22日，观察组中位无进展生存时间（progression-free survival, PFS）（7.17个月）长于对照组（4.43个月)（*P*=0.027）。观察组1年无进展生存率为15.6%，对照组为7.7%，差异无统计学差异（*P*=0.205）。治疗期间两组不良反应多为1级-2级，均可耐受，观察组患者骨髓抑制发生率高于对照组（*P* < 0.05），余不良反应与对照组无统计学差异。Cox回归分析结果显示体能状态（performance status, PS）（*P*=0.020）及年龄（*P*=0.049）是多西他赛联合PD-1/PD-L1抑制剂治疗效果的独立预后因素。

**结论:**

驱动基因阴性Ⅳ期NSCLC患者应用多西他赛联合PD-1/PD-L1抑制剂二线治疗可提高疾病控制率，延长无进展生存时间，不良反应可耐受。

## Abstract (en)

**Background and objective:**

Programmed cell death 1 or programmed cell death ligand 1 inhibitor (PD-1/PD-L1 inhibitor) and docetaxel, as the standard second-line treatments of advanced non-small cell lung cancer (NSCLC) patients, have limited effects. There are few studies on whether docetaxel combined with PD-1/PD-L1 inhibitor can increase the efficacy and make patients better benefit. The aim of this study is to evaluate the efficacy and safety of docetaxel combined with PD-1/PD-L1 inhibitor for the second-line treatment of stage Ⅳ NSCLC patients.

**Methods:**

Stage Ⅳ NSCLC patients (*n*=118) who received treatment at Shandong Cancer Hospital between October 1, 2018, and December 31, 2020, were retrospectively analyzed. They were divided into observation group (*n*=69) and control group (*n*=49) according to different treatment plan. Observation group was given docetaxel plus PD-1/PD-L1 inhibitor, while control group was given PD-1/PD-L1 inhibitor. The clinical curative effect and the incidence of adverse reactions of grade 3 and above were compared between the two groups.

**Results:**

The disease control rate (DCR) was higher in the observation group (89.9%) than that in the control group (73.5%) (*P*=0.019), and the objective response rate (ORR) showed no significant difference between observation group (24.6%) and control group (16.3%) (*P*=0.276). Till June 22, 2021, the 1-year PFS rate showed no difference between observation group (16.5%) and control group (7.7%) (*P*=0.205). During the treatment period, the adverse reactions of the two groups were mostly grade 1 to 2, and could be tolerated. The incidence of bone marrow suppression in observation group was higher than that in the control group (*P* < 0.05), and the remaining adverse reactions were not statistically different from control group. Cox regression analysis showed that performance status (PS) (*P*=0.020) and age (*P*=0.049) were independent prognostic factors for the effect of docetaxel combined with PD-1/PD-L1 inhibitor.

**Conclusion:**

The second-line treatment with docetaxel plus PD-1/PD-L1 inhibitor in patients with stage Ⅳ NSCLC can improve the DCR and prolong the PFS, and the adverse reactions are tolerable.

2020年全球癌症数据显示，肺癌发病率排名第二，占癌症总发病率的11.4%，死亡率占癌症总死亡人数的18.0%，是发病率和死亡率最高的肿瘤之一^[1]^。驱动基因阴性的晚期非小细胞肺癌（non-small cell lung cancer, NSCLC）的一线标准治疗方案已经很成熟，但二线标准治疗至今为止仅有多西他赛/培美曲塞或程序性死亡受体1（programmed cell death 1, PD-1）/程序性死亡配体1（programmed cell death ligand 1, PD-L1）抑制剂。然而无论是多西他赛/培美曲塞单药还是PD-1/PD-L1抑制剂，对经治的驱动基因阴性的晚期NSCLC患者总体有效率均不到20%，于是为了增加疗效，联合用药显得尤为重要。相关研究显示，化疗药物可以驱动免疫原性细胞死亡，激活机体适应性免疫，这种适应性免疫反应既与免疫记忆的建立相关，又具有通过γ干扰素（interferon γ, IFN-γ）依赖机制根除化疗后存活的恶性细胞的潜力；此外，化疗也可诱导亚克隆新抗原的产生，有助于增加肿瘤突变，从而激活细胞免疫反应并增强免疫检查点抑制剂的敏感性，因此，化疗药物在杀伤肿瘤细胞的同时，能够使得肿瘤患者产生功能较好的淋巴细胞，增强机体抗肿瘤免疫应答能力^[2-4]^。化疗药物与PD-1/PD-L1抑制剂在抗肿瘤治疗中具有相辅相成的作用。大量针对化疗药物联合PD-1/PD-L1抑制剂用于无驱动基因突变的晚期NSCLC一线治疗的研究^[5-9]^表明，含铂双药化疗联合PD-1/PD-L1抑制剂在无驱动基因突变的晚期NSCLC的一线治疗的疗效确切，患者耐受尚可。因此含铂双药化疗联合PD-1/PD-L1抑制剂成为驱动基因阴性的晚期NSCLC一线治疗的标准治疗方案。基于化疗药物联合PD-1/PD-L1抑制剂在晚期NSCLC患者的一线治疗取得的令人瞩目的成绩，本研究回顾分析了多西他赛联合PD-1/PD-L1抑制剂二线治疗驱动基因阴性的Ⅳ期NSCLC患者的临床疗效及安全性。

## 1 对象与方法

### 1.1 一般资料

选取2018年10月1日-2020年12月31日山东省肿瘤医院收治的符合入组标准的驱动基因阴性、一线治疗失败且驱动基因阴性的Ⅳ期NSCLC患者118例。纳入标准：①经组织学或细胞学检查确诊为Ⅳ期腺癌或鳞癌；②年龄≥18岁；③经一线治疗后病情进展；④体能状态（performance status, PS）评分≤1分；⑤表皮生长因子受体（epidermal growth factor receptor, *EGFR*）、间变性淋巴瘤激酶（anadegenerative lymphoma kinase, *ALK*）、鼠类肉瘤病毒癌基因（Kirsten rat sarcoma viral oncogene, *KRAS*）、间质-上皮细胞转化因子（mesenchymal-epithelial transition factor, *MET*）等基因突变阴性；⑥临床资料和随访资料完整。排除标准：①临床分期为Ⅰ期-Ⅲ期；②病理分型为小细胞肺癌、大细胞肺癌或者混合癌；③一线治疗方案中包含多西他赛以及PD-1/PD-L1抑制剂；④合并严重的基础性疾病或合并风湿性心脏病或肝肾功能不全或哮喘等疾病；⑤合并其他恶性肿瘤或免疫系统、血液系统疾病。按照治疗方法不同将患者分为观察组（*n*=69）和对照组（*n*=49），观察组患者接受多西他赛联合PD-1/PD-L1抑制剂治疗，对照组患者接受PD-1/PD-L1抑制剂单药治疗。分析两组患者性别、年龄、吸烟史、饮酒史、病理类型及PS评分。患者接受多西他赛联合PD-1/PD-L1抑制剂或者PD-1/PD-L1抑制剂单药治疗直至病情进展、不可耐受的毒性反应或其他原因导致停药；根据实体瘤疗效评价标准1.1（Response Evaluation Criteria in Solid Tumors 1.1, RECIST 1.1）标准，治疗期间至少每6周进行1次影像学评估[胸部计算机断层扫描（computed tomography, CT）、骨扫描、正电子发射断层扫描-计算机断层扫描（positron emission tomography-computed tomography, PET-CT）加或不加头颅磁共振成像（magnetic resonance imaging, MRI）]，整个治疗过程中不良反应进行监测。

### 1.2 治疗方法

根据治疗方法的不同将118例患者分为观察组（*n*=69）和对照组（*n*=49），对照组给予PD-1/PD-L1抑制剂治疗，包括信迪利单抗（22例）、纳武力尤单抗（12例）、帕博利珠单抗（5例）、卡瑞丽珠单抗（3例）、阿替利珠单抗（7例），静脉输液，第1天，纳武力尤单抗每2周为1个周期，其余每3周为1个周期。观察组给予PD-1/PD-L1抑制剂联合多西他赛治疗，多西他赛（江苏恒瑞医药股份有限公司，国药准字20020543，20 mg/支）75 mg/m^2^，静脉输液，第1天，每3周为1个周期；PD-1/PD-L1抑制剂包括信迪利单抗（40例）、纳武力尤单抗（12例）、帕博利珠单抗（6例）、卡瑞丽珠单抗（8例）、特瑞普利单抗（3例）。静脉输液，纳武力尤单抗每2周为1个周期，其余每3周为1个周期。用药期间定期复查血常规、肝肾功能、甲状腺功能、心肌酶谱、心电图，不良反应延迟治疗不超过7天。每6周行CT、PET-CT、全身骨扫描加或不加颅脑MRI检查进行评价疗效，用药至疾病进展或不良反应不能耐受。

### 1.3 疗效评价

应用RECIST 1.1作为参考标准，分为完全缓解（complete response, CR）、部分缓解（partial response, PR）、病情稳定（stable disease, SD）和病情进展（progressive disease, PD）。客观缓解率（objective response rate, ORR）为CR与PR之和在该组患者总例数所占的百分比，疾病控制率（disease control rate, DCR）为CR、PR与SD之和在该组患者总例数所占的百分比。无进展生存期（progression-free survival, PFS）定义为二线治疗开始至疾病进展、死亡或者患者因严重不良反应终止用药的时间。采用电话或门诊方式对患者进行定期随访，每月随访1次，随访截止时间为2021年6月22日。根据不良事件通用术语标准5.0版（Common Terminology Criteria for Adverse Events 5.0, CTCAE 5.0）来评估两组患者不良反应的发生情况。

### 1.4 统计学方法

采用SPSS 19.0软件对数据进行统计分析，计数资料以例数和率表示，组间比较采用*χ*^2^检验；*Cox*比例风险模型分析基线临床特征对患者多西他赛联合PD-1/PD-L1抑制剂治疗疗效的影响；采用*Kaplan-Meier*法绘制生存曲线，*Log-rank*检验进行单因素分析，以*P* < 0.05为差异有统计学意义。

## 2 结果

本次研究共纳入118例符合入组标准的患者，其中观察组男性44例，女性25例，对照组男性38例，女性11例，年龄18岁-75岁，如表 1所示，两组患者在性别、年龄、吸烟史、饮酒史、病理类型及PS评分均无显著差异（*P*>0.05），具有可比性。

**表 1:** 两组患者临床特征比较 Comparison of the clinical characteristics of the two groups

Characteristics		Observation group (*n*=69)	Control group (*n*=49)	*χ* ^2^	*P*
Gender	Male	44 (63.8%)	38 (77.6%)	2.567	0.109
	Female	25 (36.2%)	11 (22.4%)		
Age(yr)	≤65	52 (75.4%)	32 (65.3%)	1.413	0.235
	>65	17 (24.6%)	17 (34.7%)		
Smoking history	Yes	42 (60.9%)	30 (61.2%)	0.002	0.969
	No	27 (39.1%)	19 (38.8%)		
Drinking history	Yes	24 (34.8%)	19 (38.8%)	0.197	0.657
	No	45 (65.2%)	30 (61.2%)		
Histology	Squamous	46 (66.7%)	27 (55.1%)	1.624	0.202
	Adenocarcinoma	23 (33.3%)	22 (44.9%)		
Performance status	0	37 (53.6%)	31 (63.3%)	1.091	0.296
	1	32 (46.4%)	18 (36.7%)		

### 2.1 近期疗效

如表 2所示，观察组和对照组的ORR分别为24.6%（17/69）和16.3%（8/49），差异无统计学意义（*χ*^2^ =1.185, *P*=0.276）；观察组患者的DCR为89.9%（62/69），高于73.5%（36/49），差异有统计学意义（*χ*^2^=5.465, *P*=0.019）。

**表 2:** 两组近期疗效对比 The short-term efficacy comparison between the two groups

Group	CR	PR	SD	PD	ORR	DCR
Observation group (*n*=69)	0 (0.0%)	17 (24.6%)	45 (65.2%)	7 (10.1%)	24.6%	89.9%
Control group (*n*=49)	0 (0.0%)	8 (16.3%)	28 (57.1%)	13 (26.5%)	16.3%	73.5%
*χ* ^2^					1.185	5.465
*P*					0.276	0.019
CR: complete response; PR: partial response; SD: stable disease; PD: progressive disease; ORR: objective response rate; DCR: disease control rate						

### 2.2 长期疗效

所有患者均接受至少2个周期治疗并进行随访，随访截止时间为2021年6月22日。如图 1所示，观察组中位PFS为7.17个月（95%CI: 5.99-8.35），对照组为4.43个月（95%CI: 4.02-4.84），两者比较差异有统计学意义（*P*=0.027）。观察组1年无进展生存率为15.6%，对照组1年无进展生存率为7.7%，差异无统计学差异（*P*=0.205）。我们采用*Cox*回归模型分析了年龄、性别、吸烟史、饮酒史、病理类型、PS评分等对多西他赛联合PD-1/PD-L1抑制剂治疗效果的影响。如表 3所示，性别（*P*=0.486）、吸烟史（*P*=0.613）、饮酒史（*P*=0.923）、病理类型（*P*=0.498）与多西他赛联合PD-1/PD-L1抑制剂的PFS无显著相关性，PS（*P*=0.020）和年龄（*P*=0.049）与PFS显著相关。如图 2所示，在所有患者中，年龄≤65岁患者中位PFS为7.83个月，年龄>65岁的患者中位PFS为4.35个月，差异有统计学意义（*P*=0.005, 4）；如图 3所示，PS评分为0分的患者较PS为1分的患者中位PFS明显增高（7.23个月*vs* 4.43个月，*P*=0.001, 7）。

**图 1:**
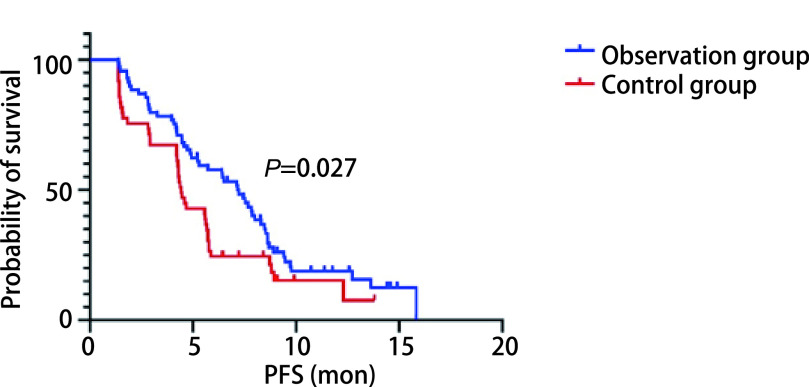
两组PFS曲线比较 Comparison of PFS curves between the two groups

**表 3:** 两组安全性比较 The safety comparison between the two groups

Toxicity	Grade 1-2		*χ* ^2^	*P*	Grade 3-4		*χ* ^2^	*P*
	Observation group (*n*=69)	Control group (*n*=49)			Observation group (*n*=69)	Control group (*n*=49)		
Bone marrow suppression	15 (21.7%)	0 (0.0%)	12.203	0.000	9 (13.0%)	0 (0.0%)	6.919	0.009
Fever	6 (4.3%)	6 (6.1%)	0.187	0.665	0 (0.0%)	0 (0.0%)	-	-
Vomiting/Diarrhea	4 (5.8%)	1 (2.0%)	0.996	0.318	4 (5.8%)	2 (4.1%)	0.175	0.676
Immune associated pneumonia	13 (18.8%)	8 (16.3%)	0.124	0.725	1 (1.4%)	2 (4.1%)	0.801	0.371
Fatigued	2 (2.9%)	0 (0.0%)	1.445	0.229	0 (0.0%)	0 (0.0%)	-	-
Liver damage	3 (4.3%)	5 (10.2%)	1.555	0.212	0 (0.0%)	0 (0.0%)	-	-
Hand-foot syndrome	1 (1.4%)	0 (0.0%)	0.716	0.397	0 (0.0%)	0 (0.0%)	-	-
Rash	2 (2.9%)	1 (2.0%)	0.078	0.780	0 (0.0%)	0 (0.0%)	-	-
Myocardial injury	0 (0.0%)	1 (2.0%)	1.420	0.233	1 (1.4%0	0 (0.0%)	0.716	0.397
Hypothyroidism	4 (5.8%)	2 (4.1%)	0.175	0.676	0 (0.0%)	0 (0.0%)	-	-

**图 2:**
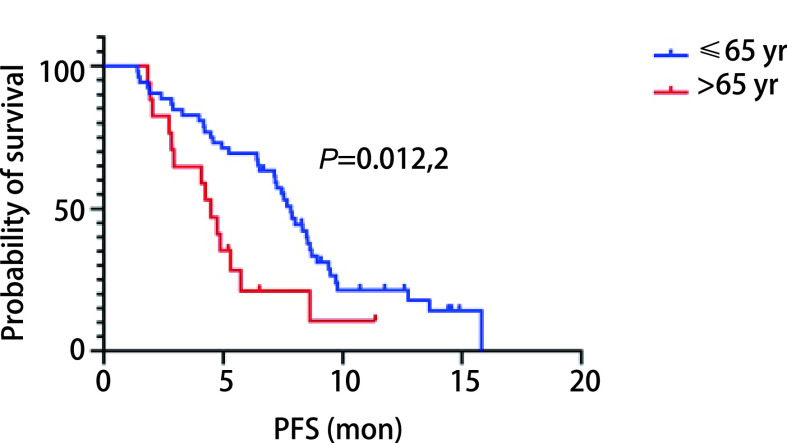
观察组患者不同年龄阶段的生存曲线 The survival curves of patients in the observation group at different ages

**图 3:**
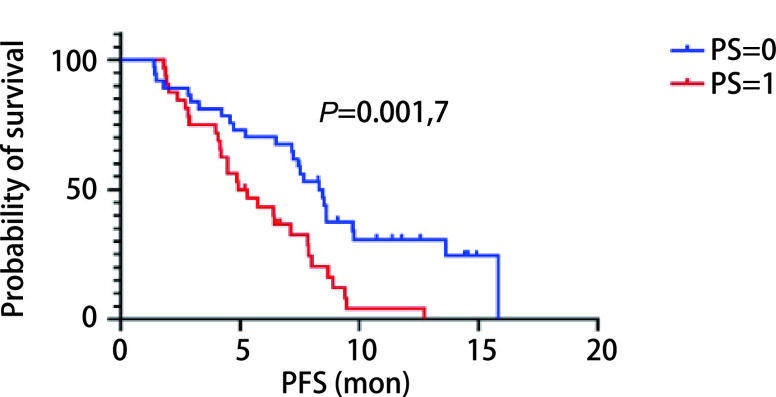
观察组患者不同体力活动状态的生存曲线 The survival curves of patients in the observation group at different performance status (PS)

### 2.3 不良反应

在我们的研究中，如表 4所示，2组患者的不良反应多数为1级-2级，患者可耐受。观察组3级-4级骨髓抑制发生率显著高于对照组（*P*=0.009），3级-4级免疫相关性肺炎（*P*=0.371），胃肠道反应（*P*=0.676）及心脏损伤（*P*=0.397）较对照组无显著差异，分别予以暂停应用PD-1/PD-L1抑制剂及应用保护胃肠道黏膜药物、升白细胞药物及激素治疗后好转。

**表 4:** 观察组*Cox*多因素分析 *Cox* multivariate analysis of the observation group

Characteristics	*P*	HR	HR (95%CI)
Gender	0.486	1.313	0.610-2.829
Age (yr)	0.049	0.471	0.222-0.996
Smoking history	0.613	0.831	0.407-1.700
Drinking history	0.923	1.034	0.526-2.034
Histology	0.498	1.276	0.631-2.579
Performance status	0.020	0.449	0.228-0.881

## 3 讨论

肺癌是中国乃至全世界发生率及死亡率最高的恶性肿瘤之一，其中非小细胞肺癌占所有肺癌病理类型的85%^[10]^。化疗、靶向及免疫治疗作为晚期NSCLC三大治疗手段，在晚期NSCLC的一线治疗中发挥着重要作用。PS评分为0分-2分的晚期NSCLC在一线治疗后继续行抗肿瘤治疗仍能从中获益，然而晚期NSCLC二线治疗方案很有限，仅PD-1/PD-L1抑制剂或多西他赛/培美曲塞单药化疗，但是实际效果并不理想。临床上亟待更加高效低毒的方案来提高患者的生存时间和生活质量。

多西他赛是肺癌中常用的化疗药物之一，由非细胞毒性前体半合成得到的类紫杉醇，能够缩短集合的滞后时间，促进微管组装成稳定的微管，提高微管组装的速度和程度，使得癌细胞周期终止于G_2_期和M期，从而抑制癌细胞有丝分裂和增殖^[11]^。2002年，席勒等^[12]^通过对比多西他赛单药与最佳支持治疗在一线或多线含铂化疗失败且PS评分为0分-2分的Ⅲb期/Ⅳ期NSCLC，结果显示多西他赛患者的进展时间比最佳支持治疗的患者更长（10.6周*vs* 6.7周，*P* < 0.001），中位总生存期（overall survival, OS）也更长（7.0个月*vs* 4.6个月，*P*=0.047），证明对于一线或多线治疗失败、PS评分在0分-2分的晚期NSCLC患者进行积极的治疗仍能使患者从中获益。Fossella等^[13]^将多西他赛100 mg/m^2^（D100）或75 mg/m^2^（D75）与长春瑞滨或异环磷酰胺（V/I）治疗既往接受过含铂治疗的晚期NSCLC患者进行比较，结果显示D100组和D75组的ORR分别为10.8%和6.7%，分别显著高于V/I的0.8%。接受多西他赛治疗的患者26周PFS更高（*P*=0.005）。D75组的1年生存率显著高于对照组（32% *vs* 19%, *P*=0.025）。Hanna等^[14]^对比培美曲塞与多西他赛在既往接受过含铂双药化疗的晚期NSCLC疗效与安全性，结果显示：培美曲塞与多西他赛ORR分别为9.1%和8.8%（*P*=0.105），中位PFS均为2.9个月，中位OS分别为8.3个月和7.9个月。自此，多西他赛/培美曲塞成为晚期NSCLC二线治疗的标准方案。

随着肿瘤分子检测水平的进步，免疫治疗一经出现便成为研究的热点，不同免疫检查点抑制剂被陆续生产并上市。用于晚期NSCLC的免疫检查点抑制剂包括PD-1/PD-L1抑制剂、细胞毒性T淋巴细胞抗原4（cytotoxic T lymphocyte associated antigen-4, CTLA-4）抑制剂、淋巴细胞激活因子-3（lymphocyte activation gene-3, LAG-3）抗体及T细胞免疫球蛋白及ITIM结构域蛋白（T cell immunoglobulin and ITIM domain protein, TIGIT）抗体等，其中PD-1/PD-L1抑制剂最常用。肿瘤细胞表达的PD-L1与T细胞表面的PD-1相结合使得T细胞受体（T cell receptor, TCR）信号通路的磷酸化，降低TCR通路下游的激活信号及T细胞的激活和细胞因子的生成。PD-1/PD-L1抑制剂能够阻断PD-1通路使得T细胞恢复活性，还能增强免疫应答，从而杀伤肿瘤细胞和阻止肿瘤细胞增殖、转移^[15]^。PD-1抑制剂纳武力尤单抗对晚期NSCLC患者的两项Ⅲ期临床研究CheckMate017和CheckMate057均表明：在鳞状或非鳞状NSCLC混合人群中，与多西他赛相比，纳武力尤单抗显示持续的OS获益，估计3年OS分别为17%（95%CI: 14%-21%）和8%（95%CI: 6%-11%），纳武力尤单抗组和多西他赛组的3年PFS分别为10%和 < 1%（HR=0.80, 95%CI: 0.69-0.92）^[16]^。因此，纳武力尤单抗对晚期NSCLC患者人群二线治疗具有长期的OS和PFS获益。吴一龙等^[17]^开展了一项纳武力尤单抗对比多西他赛在既往含铂化疗失败、驱动基因阴性的Ⅲb期/Ⅳ期或者复发的鳞状及非鳞状NSCLC患者的疗效及安全性研究（CheckMate-078研究），结果显示：无论PD-1表达如何，纳武力尤单抗组较多西他赛组显著延长OS（12.0个月*vs* 9.6个月，*P*=0.000, 6），1年生存率分别为50%和39%，ORR得到显著提高（16.6% *vs* 4.2%, *P* < 0.000, 1）。因此，2018年国家药品监督管理局（National Medical Products Administration, NMPA）批准纳武利尤单抗在NSCLC的二线适应证。KeyNote010研究^[18]^是一项随机、对照、多中心的Ⅱ期/Ⅲ期临床研究，纳入人群为PD-L1表达阳性且既往接受过至少一种化疗方案的局部晚期或转移性NSCLC患者，研究结果显示，中位OS在帕博利珠单抗2 mg/kg组、帕博利珠单抗10 mg/kg组和多西他赛组分别为10.4个月（*P*=0.000, 8）、12.7个月和8.5个月（*P* < 0.000, 1），结果均有显著差异。而在中位PFS上，帕博利珠单抗2 mg/kg组、帕博利珠单抗10 mg/kg组和多西他赛组分别为3.9个月、4.0个月和4.0个月，对比多西他赛组结果无显著差异。在亚组PD-L1≥50%的患者中，帕博利珠单抗2 mg/kg组的中位PFS明显长于多西他赛组（5.0个月*vs* 4.1个月，*P*=0.000, 1），帕博利珠单抗10 mg/kg组的中位PFS明显高于多西他赛组（5.2个月*vs* 4.1个月，*P* < 0.000, 1）。因此，美国国立综合癌症网络（National Comprehensive Cancer Network, NCCN）指南指出，帕博利珠单抗仅用于PD-L1表达水平≥1%的NSCLC患者。OAK亚组分析结果显示，阿替利珠单抗与多西他赛二线治疗晚期肺鳞癌在ORR和PFS并没有差异，而接受阿替利珠单抗治疗的患者在OS上显著获益^[19]^。综上所述，在不考虑PD-L1表达的情况下，PD-1/PD-L1抑制剂单药二线治疗驱动基因阴性的晚期NSCLC患者，可显著增加患者中位OS，但在PFS上疗效并不确切。

在本研究中，我们回顾性分析随访118例一线治疗进展的驱动基因阴性的Ⅳ期NSCLC患者的病历资料，根据治疗方法的不同，我们将其分为观察组和对照组。观察组接受多西他赛联合PD-1/PD-L1抑制剂治疗，对照组接受PD-1/PD-L1抑制剂单药治疗。观察组与对照组在性别、年龄、吸烟史、饮酒史、病理类型、PS评分等一般特点上具有可比性（*P*>0.05）。在近期疗效分析上，观察组和对照组ORR分别为24.6%和16.3%，差异无统计学意义（*χ*^2^ =1.185, *P*=0.276）；观察组患者的DCR为89.9%，显著高于对照组的73.5%，差异有统计学意义（*χ*^2^=5.465, *P*=0.019）。我们的研究结果还显示，观察组的中位PFS为7.17个月（95%CI: 5.99-8.35），对照组为4.43个月（95%CI: 4.02-4.84），两者比较差异有统计学意义（*P*=0.027）。观察组1年无进展生存率为15.6%，对照组1年无进展生存率为7.7%，差异无统计学意义（*P*=0.205）。由此可见，在不考虑PD-L1表达的前提下，多西他赛联合PD-1/PD-L1抑制剂对比PD-1/PD-L1抑制剂单药二线治疗Ⅳ期NSCLC能使患者在DCR和PFS上明显获益，但是在ORR和1年无进展生存率上疗效并不显著。

随着中国人口的不断增加及老龄化，老年肺癌患者的发病率也日渐升高，对于60岁以上的老年患者，肺癌是主要的癌性死亡原因^[20]^。老年人肺癌发生率高的原因主要在于虚拟记忆T细胞（virtual memory T cell, TVM）会随着年龄的增长而增多，TVM缺乏对TCR信号作出反应的增殖能力，使得CD8^+^ T淋巴细胞出现与年龄相关的增殖缺陷，同时，其他功能性T淋巴细胞的增殖受到TVM的阻碍，从而导致幼稚T淋巴细胞减少^[21]^。Zhang等^[22]^认为，在年龄分界线为65岁时，抗PD-1/PD-L1抑制剂对年轻及老年患者均有效，在年轻患者的疗效（HR=0.75, 95%CI: 0.65- 0.87）略高于老年患者（HR=0.81, 95%CI: 0.72-0.92）。在年龄分界线为75岁时，PD-1/PD-L1抑制剂在老年患者中的获益不明显。随着年龄的增长，患者的身体机能不断下降Wedding等^[23]^认为，虽然老年晚期非小细胞肺癌患者化疗的不良反应较大，但是当PS作为评价疗效的指标时，年龄则显得没那么重要。在本研究中，以65岁为分界线，观察组年龄>65岁患者有16例，与该组年龄≤65岁患者相比，老年患者的中位PFS缩短了3.36个月（4.47个月*vs* 7.83个月），差异有统计学意义（*P*=0.012, 2）。因此，年龄可被认为是影响多西他赛联合PD-1/PD-L1抑制剂治疗效果的独立预后因素，年龄越小，患者获益越多。结合检索到的相关文献可知，在年龄≥75岁的晚期NSCLC患者二线治疗应用PD-1/PD-L1抑制剂效果不佳，因此我们有理由慎重考虑在年龄≥75岁的晚期NSCLC患者二线是否应用多西他赛联合PD-1/PD-L1抑制剂治疗。

此外，PS评分也是影响多西他赛联合PD-1/PD-L1抑制剂治疗效果的独立预后因素。本研究结果显示，当PS评分为0分时，患者的中位PFS为8.30个月，当PS评分为1分时，患者的中位PFS为5.12个月，差异有统计学意义（*P*=0.001, 7）。当患者PS评分较低时，患者的身体条件较好，对PD-1/PD-L1抑制剂有良好的耐受性^[24]^。因此，患者PS评分越低，对药物的耐受性越好，应用多西他赛联合PD-1/PD-L1抑制剂治疗效果越好。

综上所述，多西他赛联合PD-1/PD-L1抑制剂能够给一线治疗进展、驱动基因阴性的Ⅳ期NSCLC患者带来良好的治疗效果，在DCR以及PFS更好的获益，且毒副反应低，安全可控。但是由于我们的样本量太小，免疫药物使用较杂且随访时间较短，无法针对PD-1/PD-L1抑制剂进行单独对比，也未能对PD-L1表达情况进行分组和统计OS的结果。因此后续我们会继续扩大样本量，进一步分析在不同PD-L1表达和不同的PD-1/PD-L1抑制剂药物使用情况下多西他赛联合PD-1/PD-L1抑制剂的二线治疗效果，以及继续随访统计该方案二线治疗驱动基因阴性的晚期NSCLC的OS结果。
